# Are one or two simple questions sufficient to detect depression in cancer and palliative care? A Bayesian meta-analysis

**DOI:** 10.1038/sj.bjc.6604396

**Published:** 2008-05-27

**Authors:** A J Mitchell

**Affiliations:** 1 Department of Cancer & Molecular Medicine, Leicester Royal Infirmary, Leicester LE1 5WW, UK; 2 Leicester General Hospital, Leicester LE5 4PW, UK

**Keywords:** depression, diagnostic validity, meta-analysis, sensitivity

## Abstract

The purpose of this study is to examine the value of one or two simple verbal questions in the detection of depression in cancer settings. This study is a systematic literature search of abstract and full text databases to January 2008. Key authors were contacted for unpublished studies. Seventeen analyses were found. Of these, 13 were conducted in late stage palliative settings. (1) Single depression question: across nine studies, the prevalence of depression was 16%. A single ‘depression’ question enabled the detection of depression in 160 out of 223 true cases, a sensitivity of 72%, and correctly reassured 964 out of 1166 non-depressed cancer sufferers, a specificity of 83%. The positive predictive value (PPV) was 44% and the negative predictive value (NPV) 94%. (2) Single interest question: there were only three studies examining the ‘loss-of-interest’ question, with a combined prevalence of 14%. This question allowed the detection of 60 out of 72 cases (sensitivity 83%) and excluded 394 from 459 non-depressed cases (specificity of 86%). The PPV was 48% and the NPV 97%. (3) Two questions (low mood and low interest): five studies examined two questions with a combined prevalence of 17%. The two-question combination facilitated a diagnosis of depression in 138 of 151 true cases (sensitivity 91%) and gave correct reassurance to 645 of 749 non-cases (specificity 86%). The PPV was 57% and the NPV 98%. Simple verbal methods perform well at excluding depression in the non-depressed but perform poorly at confirming depression. The ‘two question’ method is significantly more accurate than either single question but clinicians should not rely on these simple questions alone and should be prepared to assess the patient more thoroughly.

There is a general consensus that it is important to recognise and treat depression during the course of cancer, especially in palliative stages where particular emphasis is on quality of life (Stiefel *et al*, 2001; Noorani and Montagnini, 2007). Distress, anxiety and depression powerfully influence quality of life as well as satisfaction with care and participation in medical treatment (Skarstein *et al*, 2000; Stark *et al*, 2002; Kennard *et al*, 2004; Bui *et al*, 2005). Studies that have used structured psychiatric interviews suggest that the median prevalence of major depressive disorder is 15% in advanced cancer (Hotopf *et al*, 2002). Four large-scale studies using severity scales suggest that the overall prevalence of distress in unselected cancer patients is above 30% (Pascoe *et al*, 2000; Fallowfield *et al*, 2001; Zabora *et al*, 2001; Carlson *et al*, 2004). Yet, it is well known that syndromal anxiety and depression are often overlooked by busy cancer professionals in palliative and non-palliative settings (Ford *et al*, 1994; Fallowfield *et al*, 2001; Sollner *et al*, 2001; Stiefel *et al*, 2001; Durkin *et al*, 2003; Sharpe *et al*, 2004) and the majority of patients will not gain access to mental health services (Kadan-Lottick *et al*, 2005). In part, this is because cancer specialists have difficulty in identifying emotional complications and tend to have communication behaviours that systematically focus on physical rather than psychological concerns (Durkin *et al*, 2003).

More than 50 questionnaires have been developed to aid the detection of depression or severe distress, but most have been validated in primary care rather than cancer settings (Lloyd-Williams *et al*, 2003b). Perhaps, best known of all depression scales is the Patient Health Questionnaire (in either nine or two item forms) (Spitzer *et al*, 1999). This scale has some merit in primary care and appears highly acceptable (Mitchell and Coyne, 2007) but has yet to be rigorously tested in cancer settings. Indeed, only a handful of tools have been studied specifically in palliative care (Le Fevre *et al*, 1999; Holtom and Barraclough, 2000; Lloyd-Williams *et al*, 2001, 2004; Love *et al*, 2004; Thekkumpurath *et al*, 2007). Their main limitation, however, is that they are often too long for routine use (Mitchell *et al*, 2008). In response to this problem, short versions of many common depression scales have been developed. These include 7 and 6 item versions of the Hamilton Depression Rating Scale (HAMD) (McIntyre *et al*, 2005; Guo *et al*, 2006; Serrano-Duen and Soledad, 2008); 13, 7 and 2 item versions of the Beck Depression Inventory (BDI) (Steer *et al*, 1999; Furlanetto *et al*, 2005; Huffman *et al* 2006); 13, 10 and 6 item versions of the Center for Epidemiologic Studies Depression Scale (CES-D) (Burnam *et al*, 1988; Cole *et al*, 2004; Covic *et al*, 2007); 5, 4 and 2 item versions of the Geriatric Depression Scale (GDS) (Andresen *et al*, 1994; van Marwijk *et al*, 1995; Hoyl *et al*, 1999); and 8, 6 and 5 item versions of the Edinburgh Depression scale (EPDS) (Pallant *et al*, 2006; Eberhard-Gran *et al*, 2007; Lloyd-Williams *et al*, 2007). Occasionally, authors have developed entirely new short scales, such as the four item case-find for depression (Jefford *et al*, 2004), or attempted to develop a short scale specifically for palliative settings (Lloyd-Williams *et al*, 2007).

In the 1990s, several groups working in cancer settings suggested that two questions, or in some cases just a single question, might be sufficient to detect depression in palliative care. Usually, these ultra-short tests formed part of symptom checklists and were not validated against an accepted standard (Miller and Walsh, 1991; Donnelly *et al*, 1995; Conill *et al*, 1997; Brunelli *et al*, 1998; Edmonds *et al*, 1998; Ng and von Gunten, 1998; Pratheepawanit *et al*, 1999). At the same time, simple (non-verbal) visual-analogue methods of assessing depression, anxiety or distress were developed, exemplified by the NCCN Distress Thermometer and Edmonton Symptom Assessment (Hürny *et al*, 1996; Vignaroli *et al*, 2006). The accuracy of these methods was reviewed in mixed cancer settings with the finding that they had reasonable rule-out accuracy but limited case-finding ability (Mitchell, 2007). Yet, it is not clear how simple verbal questions perform alone and when used specifically for patients with advanced cancer.

The aim of this study is to examine the diagnostic accuracy of simple verbal questions to detect depression in cancer and palliative care and to ascertain whether clinicians should rely upon either one question or two questions to detect major depression compared with more established screening tools.

## MATERIALS AND METHODS

### Search

A systematic literature search, critical appraisal of the collected studies and pooled analysis were conducted. The following abstract databases were searched: Medline 1966-January 2008, PsycINFO 1887-January 2008, Embase 1980-January 2008 and CINAHL 1982-January 2008. In these databases, the keywords (MeSH terms) were ‘distress or anxi$ or depress$ or mood’ and ‘screen$ or detect$ or case-finding or recogni$ or diagnos$ or recogni$’ and ‘cancer or oncology or malignant or transplant or tumour or metastatic.’ Four full text collections including Science Direct, Ingenta Select, Ovid Full text and Wiley Interscience were searched. In these online databases, the same search terms were used but as a full text search and citation search. The abstract database Web of Knowledge (4.0, ISI) was searched, using the above terms as a text word search, and using key papers in a reverse citation search. Conference abstracts from IPOS 2006 and 2007 were examined. Non-English language papers and abstracts were included but, where necessary, authors were contacted directly for primary data and data in press.

### Critical appraisal

The review guidelines for diagnostic tests recently outlined in Evidence Based Medicine were followed (Pai *et al*, 2004). Questions for each report included the setting, the data integrity, the choice of reference criterion, the method of application of the screening questionnaire and, importantly, the type of outcome measured. Quality appraisal standards are listed in Table 1.

### Pooled analysis and meta-analysis

Two methods are possible in combining diagnostic validity studies (Midgette *et al*, 1993; Irwig *et al*, 1995). (a) Simple pooling of the raw data and re-calculation of the cumulative sensitivity, specificity, positive predictive value (PPV) and negative predictive value (NPV). This method assumes a consistent prevalence between studies and in future work. (b) Correction for the variance in prevalence by relying on the stability of sensitivity and specificity by calculating a pooled weighted rate of sensitivity and specificity and then calculating PPV and NPV according to local prevalence data (Glasziou and Irwig, 1998). In this case, a Bayesian curve can be constructed of all post-test probabilities if a given test is positive or negative. Overall accuracy was calculated using the identificiation index which is the fraction correct minus faction incorrect. The reciprocal of the identification index is the number needed to screen (Mitchell, 2009).

### Standards of accuracy

Performance was taken as follows: <0.2 poor, >0.2⩽0.4 fair, >0.4⩽0.6 moderate, >0.6⩽0.8 good and >0.8⩽1 very good; adapted from that originally proposed by Landis and Koch (Landis and Koch, 1977).

### Outcome measures

The majority of studies defined depression using a psychiatric interview (applied in a semistructured or clinical interview) but a minority utilised standardised rating scales (Murphy, 2002).

## RESULTS

### Systematic literature search

**Figure 1:**
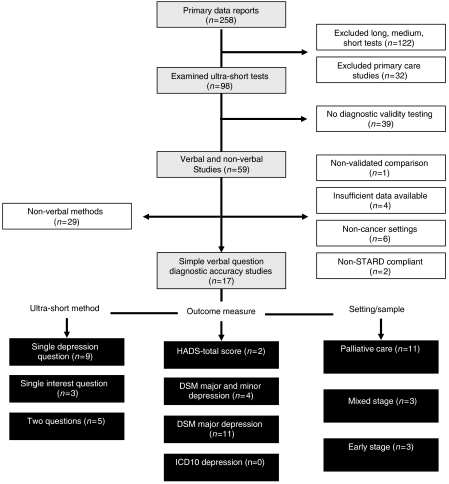
Quorom diagram of studies.

The search identified 98 analyses specifically examining ultra-short methods (Figure 1). Studies that examined one or two question methods in non-cancer medical patients or in primary care were excluded. A total of 39 studies had no gold standard and 29 examined visual-analogue methods and were therefore excluded. Thirteen studies were not sufficiently detailed for inclusion. Thus, 17 analyses of verbal/written questions to detect depression in cancer were included. No attempt was made to separate questions read by investigators (verbal) from questions read by the patients (written). The data extraction is illustrated in Figure 1 in accordance with Quality of Reporting of Meta-analyses guidelines (Moher *et al*, 1999).

Of 17 analyses, 9 examined one single depression question: Chochinov *et al* (1997); Lloyd-Williams *et al* (2003a); Meyer *et al* (2003a); Jefford *et al* (2004); Akechi *et al* (2006); Kawase *et al* (2006); Ohno *et al* (2006); Payne *et al* (2007); and Mitchell *et al* (2008). Three analyses examined one single interest item: Akechi *et al* (2006); Payne *et al* (2007); and Mitchell *et al* (2008). Five analyses examined a combination of two questions: Akechi *et al* (2006); 65 Payne *et al* (2007); Chochinov *et al* (1997); Gessler *et al* (2007); and Mitchell *et al* (2008).

### Critical appraisal

The mean sample size was 165.8 (s.d. 55.7). However, several studies examined different verbal methods in the same sample and thus there were actually 1579 unique patients under study. Eleven studies took place in palliative settings and/or specifically in those with late-stage cancer. Three took place in mixed stages and three in predominantly early cancers. All but two studies used DSM criteria by clinical interview or by structured clinical interview. Four looked at major or minor depression combined and the remainder looked at major depression alone.

The most common question for depression was simply ‘Are you depressed?’ but variations included ‘Describe your mood over the last week’ and the PHQ2 question two ‘Over the last 2 weeks, how often have you been bothered by feeling down, depressed or hopeless?’ The loss of interest question was asked in three different ways, namely ‘Have you lost interest?’, ‘Have you experienced loss of interest in things or activities that you would normally enjoy?’ and the PHQ2 question one ‘Over the last 2 weeks, how often have you been bothered by little interest or pleasure in doing things?’ The two-question approach was always asked as question 1 (Q1) *or* question 2 (Q2), which favours sensitivity at the expense of specificity compared to the Q1 *and* Q2 approach (Coyne and Mitchell, 2007). Further details of the studies are shown in Tables 1 and 2.

### Pooled analysis and meta-analysis

#### Single depression/mood question

Across nine studies, the prevalence of depression was 16%. Using the simple pooled method, the single depression question enabled the detection of depression in 160 out of 223 true cases, a sensitivity of 72% (95% CI 66.3–76.8%) and correctly reassured 964 out of 1166 non-depressed cancer sufferers, a specificity of 83% (95% CI 81.6–83.6). Thus, the PPV was 44% and the NPV was 94%. The Youden score was 0.544 (95% CI 47.9–60.3). Using the meta-analytic approach, the weighted sensitivity was 74.1% (95% CI=0.68–0.80) and the specificity was 85.8% (95% CI 83.7–87.7).

**Figure 2:**
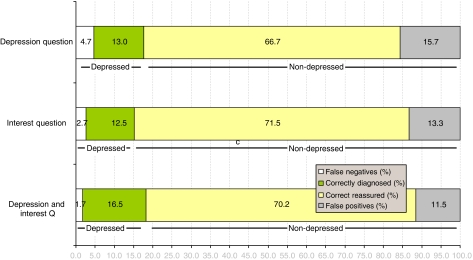
Natural screening accuracy of simple verbal questions in the detection of cancer-related depression.

Analysing the results by natural frequencies, out of every 100 screening applications, the single depression question would correctly rule out 69 non-depressed, rule in 11 out of 18 cases, missing 5 and also giving 15 false-positive diagnoses (Figure 2). Thus, the identification index (net gain) would be 61.8% and the number needed to screen in order to yield one additional correct identification would be 1.62.

#### Single interest question

There were only three studies examining the ‘loss-of-interest’ question, with a pooled prevalence of 14%. Using the simple pooled method, this question allowed the detection of 60 out of 72 cases (sensitivity 83%) (95% CI 74.2–89.9) and excluded 394 from 459 non-depressed cases (specificity of 86%) (95% CI 84.4–89.9). The PPV was 48% and the NPV 97%. The Youden score was 0.692 (95% CI 0.586–0.768). Using the meta-analytic approach, the weighted sensitivity was 82.4% (95% CI=73.0–90.0) and the specificity was 86.4 (95% CI=83.0–89.3).

Analysing the results by natural frequencies, out of every 100 screening applications, the single loss of interest question would correctly rule out 74 non-depressed, rule in 11 out of 14 cases, missing 2 and also giving 12 false-positive diagnoses (Figure 2). Thus, the identification index (net gain) would be 71% and the number needed to screen in order to yield one additional correct identification would be 1.41.

#### Two questions (low mood and low interest)

In five studies using a two-question combination (Q1 or Q2), the prevalence of depression was 17%. Using the simple pooled method, the two-question combination facilitated a diagnosis of depression in 138 of 151 true cases (sensitivity 91.4%) (95% CI 86.4–94.8) and gave correct reassurance to 645 of 749 non-cases (specificity 86%) (95% CI 85.1–86.8). The PPV was 57% and the NPV 98%. The Youden score was 0.775 (95% CI 0.771–0.816), significantly higher than either of the single questions used alone. Using the meta-analytic approach, the weighted sensitivity was 92.7% (95% CI=88.1–96.3) and specificity 87.4% (95% CI=84.9–89.7).

Analysing the results by natural frequencies, out of every 100 screening applications, these two questions would correctly rule out 72 non-depressed, rule in 15 out of 18 cases, overlooking 1 true case and also giving 12 false-positive diagnoses (Figure 2). Thus, the identification index (net gain) would be 74% and the number needed to screen in order to yield one additional correct identification would be 1.35.

#### Bayesian pre-test–post-test gain

**Figure 3:**
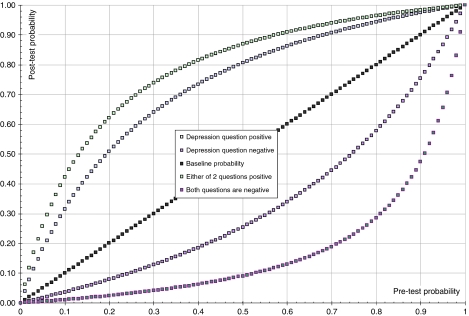
Bayesian graph: test probability (PPV and NPV) as function of test result – depression question *vs* combination question.

Assuming sensitivity and specificity hold for different rates of depression, a Bayesian curve was constructed of all post-test probabilities where a test result was either positive or negative. This illustrates the pre-test–post-test gain for each method and the predictive value conditional upon different baseline rates of depression. Figure 3 demonstrated the superior difference in gain for the two-question approach with the depression question alone.

## CONCLUSIONS

A previous pooled analysis found eight diagnostic validity analyses of one or two single item questions in the detection of depression in cancer settings (Mitchell, 2007). This study updates the previous analysis to include 17 analyses, 13 involving late-stage cancer and/or palliative settings. It is important to note that the average prevalence of depression across these studies was 16% (range 7–38%), which means that any case-finding method is likely to have difficulty detecting true cases without generating false positives. Results show that the loss of interest question is somewhat better than the depression question when used alone. This corresponds to research showing that, of many symptoms of depression, loss of interest best discriminated between patients with and without diagnosis of comorbid affective disorder (Reuter *et al*, 2004). However, two questions are significantly better than any one question for detecting depression (Youden score was 0.78 for two questions, 0.54 for the depression question and 0.69 for the interest question). In fact, two questions are better for both ruling in and ruling out a diagnosis than either question alone, although the loss of interest question is also an excellent method of excluding depression. No method achieved a case-finding accuracy of more than 60% according to the PPVs. This means that at best there would be 43 false positives out of each 100 positive screens.

There has been increasing interest in short verbal and non-verbal screening methods. Lloyd-Williams and colleagues (Lawrie *et al*, 2004) found that among consultants working in palliative medicine, 10% asked the patient ‘are you depressed’ to detect depression. Mitchell *et al* (2008) and colleagues found that simple verbal questions were the most preferred active method of detecting depression, used by 30% of cancer professionals. However, this meta-analysis raises an important caution for all those using one or two questions. Assuming use of the two-question combination, this would mean that out of every 100 screening applications answering yes to either of these two questions, only 1 true case would be missed but 12 false-positive diagnoses would be generated (Figure 2). Thus, a second method with better PPV would be required. This could be a thorough clinical assessment by someone confidently able to diagnose depression or it could be a longer validated depression severity scale (Robinson and Crawford, 2005). However, there is no agreement on which is the optimal case-finding method and rarely has any method shown a case-finding (PPV) accuracy that exceeds 0.80. (Lloyd-Williams *et al*, 2003b; Trask, 2004). There is also no agreement on how often a tool should be applied (Lloyd-Williams and Riddleston, 2002). For example, Love *et al* (2004) found that the HADS depression subscale had a PPV of 0.79 when a cut-off of 7v8 was used. Using the same cut-off, Le Fevre *et al* (1999) found a PPV of 0.42 in a palliative settings. Recently, Lloyd-Williams *et al* (2007) showed that a six-item adaptation of the EPDS had a PPV of 0.65, also in a palliative setting.

In conclusion, no method has been shown to be sufficiently accurate to be considered the definitive screening or case-finding tool for cancer-related depression. Simple questions should be considered as a method of exclusion or combined with more detailed tests. Future work should move beyond screening for psychopathology alone to also consider unmet needs. That is, those individuals with emotional disorders (distress, anxiety, depression and anger) who require and desire professional help (Graves *et al*, 2007).

## Figures and Tables

**Table 1:** Methodological aspects of simple verbal questions for depression in cancer

**Year**	**Author**	**Screening question**	**Reference Standard**	**Sample used for diagnostic testing ^(u)^**	**Setting**	**Comment**	**Quality appraisal**
1997	Chochinov *et al*, 1997	‘Are you depressed?’ ‘Are you depressed OR have you lost interest?	RDC Mj+Mn Dep	197	Palliative	Sample was 94 male and 103 female inpatients receiving palliative care for advanced terminal cancer. Enhanced reliability was attempted by having an observer (a psychiatrist or psychologist) attend a random sample of 27 interviews. Chochinov *et al* examined whether the first two questions of the diagnostic interview could be *used on their own when used at the same time* as the clinical interview. The lack of administration of a blind independent criterion standard may partly explain the high accuracy of this method alone.	Sample–Adequate Blinding–Poor Gold Standard–Adequate Sample integrity–Adequate
2003	Lloyd-Williams *et al*, 2003a	‘Are you depressed?’	Clinical interview based on DSMIV	74	Palliative	Recruited those in palliative and supportive day care over 6 months. Gold standard was the semi-structured clinical psychiatric interview based on DSMIV, although the exact method of administration was not disclosed.	Sample–Poor Blinding–Adequate Gold Standard–Adequate Sample integrity–Good
2006	Akechi *et al*, 2006	‘Are you depressed?’ ‘Have you lost interest?’	1. Structured clinical interview for DSM-III-R.Mj Dep 2. Structured clinical interview for DSM-III-R.Mj Dep+ adjustment	205	Palliative	The reliability (kappa coefficient) of the interview ratings was investigated by having another trained psychiatrist attend the first 29 consecutive interviews as a second rater. Mean age was 61 years; 137 (66%) subjects were male and 51 (24%) were in full-time employment. The most frequent primary cancer site was the lung (38%). In head-to-head analysis the single-item interview ‘Have you lost interest or pleasure?’ (AUC 0.92) performed better than ‘Are you depressed?’ (AUC 0.85) or indeed the HADS-D arm (AUC=0.82) or the HADS-Total score (AUC=0.79).	Sample–Adequate Blinding–Adequate Gold Standard–Good Sample integrity–Adequate
2006	Kawase *et al*, 2006	‘Are you depressed?’	DSM-IV-TR Mj+Mn Dep	282	Mixed Cancer Outpatients undergoing radiotherapy	Sample aged 26–90 years (mean 62.2). Interview by clinical psychologists with 100% concordance. Major and minor depression not separated in the analysis.	Sample–Good Blinding–Adequate Gold Standard–Adequate Sample integrity–Adequate
2007	Payne *et al* (2007)	‘Are you depressed?’ OR ‘Have you experienced loss of interest in things or activities that you would normally enjoy?’	Clinical interview based on DSMIV major depressive disorder as defined by DSM conducted by one of two mental health professionals	167 (74% were suffering from cancer)	Inpatient Palliative Unit	A subgroup analysis of individuals with a past experience of depressive illness, (n 95) revealed that a significant number screened positive for depression by the screening test, 55.2% (16/29) compared with those with no background history of depression, 33.3% (22/66) (*P*=0.045).	Sample–Adequate Blinding–Adequate Gold Standard–Adequate Sample integrity–Adequate
2004	Jefford *et al*, 2004	‘Over the past couple of weeks, have you been feeling unhappy or depressed?’	PRIME-MD (DSMIV) but validation based on the 4Q Brief Case-Find for Depression (BCD)	100	Late (60% palliative)	Also used Prime-MD, BDI and HADS. Patients with depression had more pain and inferior performance status/functioning. Authors suggest that the BCD can be administered in 1 min in oncology and palliative settings.	Sample–Poor Blinding–Poor Gold Standard–Poor Sample integrity–Adequate
2006	Ohno *et al*, 2006	‘Are you depressed or not?’	HADS Combined >14	160	Mixed Cancers	Assessed diagnostic accuracy of patients who refused to answer Q ‘are you depressed’	Sample–Adequate Blinding–Poor Gold Standard–Poor Sample integrity–Adequate
2003	Meyer *et al*, 2003	Schedule of affective disorders and schizophrenia (SADS) item: please describe your mood over the past week(s) (or since last seen) in terms of low mood or depression (not at all, slight *vs* mild, moderate or severe).	Structured clinical interview for DSM-III-R (SCID), a semi-structured interview for diagnosing depression	45	Inpatients with advanced cancer (prognosis B/6 months) consecutively referred to a hospital palliative care team	Authors compared mood evaluation questionnaire (MEQ) and the structured clinical interview for DSM-III-R (SCID) as well as the single-item interview screening question. The MEQ and SCID had moderate agreement (weighted kappa 0.52 over all interviews). At first interview, 26 (58%) patients were depressed using MEQ, seven (16%) of these severely.	Sample–Poor Blinding–Adequate Gold Standard–Good Sample integrity–Adequate
2007	Gessler *et al*, 2007 (online published)	2Q‘Have you lost interest or pleasure?’ OR ‘Are you depressed?’	HADS combined >11	160	Mixed outpatients with cancer	53% female and mean age 60.0 years. Also tested were the HADS, GHQ-12, BSI-18 and DT.	Sample–Adequate Blinding–Poor Gold Standard–Poor Sample integrity–Adequate
2008	Baker-Glenn, Thiagarajan; Chaudhuri, Granger, Symonds, Mitchell, 2008 (unpublished)	PHQ Q1 – ‘Over the last 2 weeks, how often have you been bothered by little interest or pleasure in doing things?’ PHQ Q2 ‘Over the last 2 weeks, how often have you been bothered by feeling down, depressed or hopeless?’	DSMIV major depression	215	Cancer patients attending chemotherapy	Mean age was 57.7 years and their mean time from diagnosis 7.3 months. The prevalence of major depressions was 11.1%. Patients also received the HADS, PHQ9 and DT.	Sample–Adequate Blinding–Poor Gold Standard–Adequate Sample integrity–Adequate

(u)=Uncorrected; 1Q=single question test, 2Q=two question test; RDC=research diagnostic criteria; HADS=Hospital Anxiety and Depression Scale; CESD=Center for Epidemiologic Studies Depression Scale (CES-D); BCD=Brief Case Find for Depression includes the items ‘(A) been having restless or disturbed nights? (B) been feeling unhappy or depressed? (C) felt unable to overcome your difficulties? (D) been dissatisfied with the way you've been doing things.’

Quality appraisal: Sample <100=poor; <250=adequate; <500=good; >499=excellent; Blinding – both scale and gold standard by same rater=poor; different rater=adequate; different rate blind to results=good; Gold Standard–Depression severity scale=poor; DSM or ICD10 criteria=adequate; semi-structured or structured interview=good; sample integrity – unexplained missing data – poor; partial loss to follow-up=adequate; no missing data=good. Dep=Depression; PHQ=Patient Health Questionnaire; Mj=Major; Mn=Minor.

**Table 2:** Statistical summary of simple verbal questions for depression in cancer

**Reference**	**Assessment of depression**	**Total cases with depression**	**Sensitivity**	**Total cases without depression**	**Specificity**	**PPV**	**NPV**	**Youden score**	**Number needed to screen^a^**
*Depression question*									
Akechi *et al*, 2006	1Q – ‘Are you depressed?’	14	0.79	195	0.92	0.41	0.98	0.70	1.22
Payne *et al*, 2007	1Q – ‘Are you depressed?’	43	0.70	124	0.81	0.57	0.89	0.51	1.76
Chochinov *et al*, 1997	1Q – ‘Are you depressed?’	24	1.00	173	1.00	1.00	1.00	1.00	1.00
Lloyd-Williams *et al*, 2003a	1Q – ‘Are you depressed?’	20	0.55	54	0.74	0.44	0.82	0.29	2.64
Jefford *et al*, 2004	1Q – Over the past couple of weeks, have you been feeling unhappy or depressed?	12	0.67	88	0.70	0.24	0.94	0.37	2.50
Ohno *et al*, 2006	1Q – ‘Are you depressed or not?’	54	0.93	106	0.31	0.41	0.89	0.24	26.67
Meyer *et al*, 2003	1Q – ‘Describe your mood over the last week’	17	0.35	28	0.75	0.46	0.66	0.10	5.00
Baker-Glenn, Thiagarajan; Chaudhuri, Granger, Symonds, Mitchell, 2008 (unpublished)	1Q – ‘Over the last 2 weeks, how often have you been bothered by feeling down depressed or hopeless?’	15	0.67	140	0.95	0.59	0.96	0.62	1.18
Kawase *et al*, 2006	1Q – ‘Are you depressed?’	24	0.42	258	0.86	0.22	0.94	0.28	1.55
**Pooled summary**	**Subtotal – depressed**	**223**	**0.72**	**1166**	**0.83**	**0.44**	**0.94**	**0.54**	**1.62**
									
*Interest question*									
Payne *et al*, 2007	1Q – ‘Have you experienced loss of interest in things or activities that you would normally enjoy?’	43	0.79	124	0.73	0.50	0.91	0.52	2.06
Baker-Glenn, Thiagarajan; Chaudhuri, Granger, Symonds, Mitchell, 2008 (unpublished)	1Q – Over the last 2 weeks, how often have you been bothered by little interest or pleasure in doing things?'	15	0.87	140	0.89	0.46	0.98	0.76	1.28
Akechi *et al*, 2006	1Q – ‘Have you lost interest?’	14	0.93	195	0.92	0.45	0.99	0.85	1.19
**Pooled summary**	**Subtotal – interest**	**72**	**0.83**	**459**	**0.86**	**0.48**	**0.97**	**0.69**	**1.41**
									
*Combination question*									
Payne *et al*, 2007	2Q – ‘Depressed or loss of interest’	43	0.91	124	0.68	0.49	0.95	0.58	2.11
Akechi *et al*, 2006	2Q – ‘Have you lost interest or pleasure?’ OR ‘Are you depressed?’	14	1.00	195	0.86	0.34	1.00	0.86	1.35
Baker-Glenn, Thiagarajan; Chaudhuri, Granger, Symonds, Mitchell, 2007 (online published)	2Q – PHQ1 or 2	15	1.00	140	0.87	0.45	1.00	0.87	1.30
Gessler *et al*, 2007 (online published)	2Q – ‘Have you lost interest or pleasure?’ OR ‘Are you depressed?’	43	0.79	117	0.86	0.68	0.92	0.65	1.45
Chochinov *et al* (1997)	2Q – Depressed mood OR loss of interest	36	1.00	173	0.98	0.92	1.00	0.98	1.03
**Pooled summary**	**Subtotal – 2Q**	**151**	**0.91**	**749**	**0.86**	**0.57**	**0.98**	**0.78**	**1.35**

PPV=positive predictive value; NPV=negative predictive value. 1Q=Single question; 2Q=Two question combination.

a Number needed to screen=reciprocal of fraction correct minus faction incorrect.
